# Supplementary figures and images for: In-vivo egfp expression in the honeybee Apis mellifera induced by electroporation and viral expression vector
Source: PLoS One. 2022 Jun 2;17(6):e0263908. doi: 10.1371/journal.pone.0263908 (PMC9162312; doi:10.1371/journal.pone.0263908)

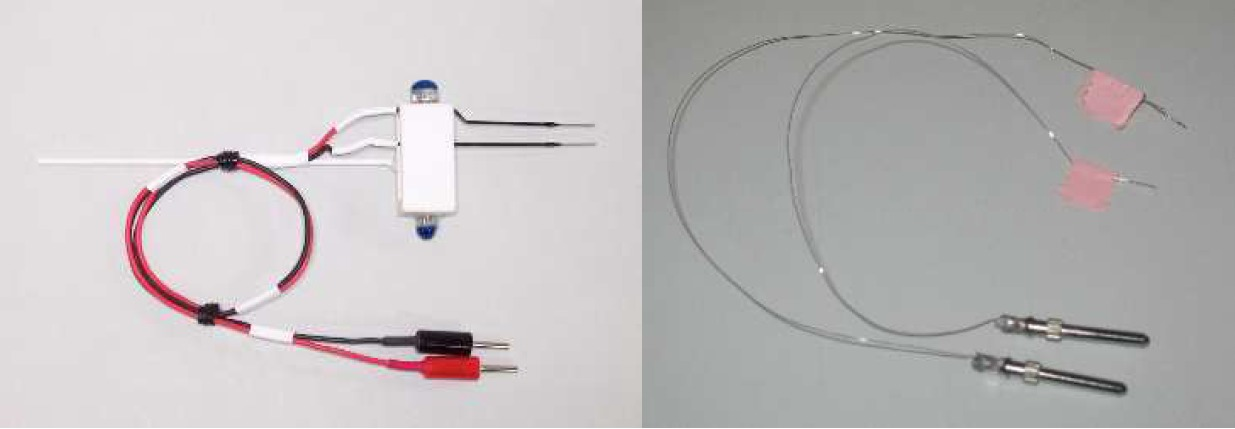

Supplement: S1 Fig — Immediately after the injection thin cuts were performed in the cuticle at the dorsal edge of each compound eye and the tip of the electrodes were inserted in the retina of each eye (Left). For the local injection protocols, a window was cut in the cuticle, adjacent tissues were put aside and the electrodes were placed at the surface of the retina (right). (TIF) [file pone.0263908.s001.tif]

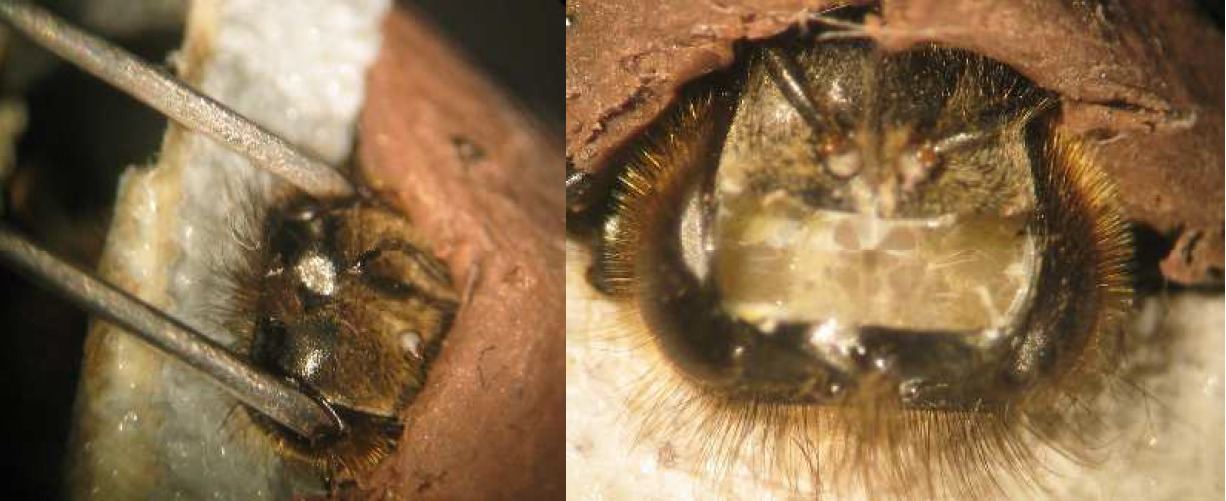

Supplement: S2 Fig — The CUY567 electrode of the manufacturer (Nepagene, Chiba, Japan; Xceltis, Mannheim, Germany)(Left). Custom made platinum electrodes of different diameters were used, here 0.125 mm (large wire electrodes) (Advent Research Materials Ltd, Witney, United Kingdom) with Teflon insulation (right). (TIF) [file pone.0263908.s002.tif]

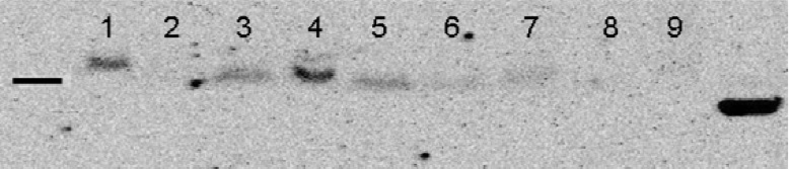

Supplement: S3 Fig — A single protein migrating, as expected, at an apparent molecular weight of 32 kDa was detected by Western blot on brains of electroporated animals, at 100 V with 5X 50 ms pulses (1–7) but not at 150 V 5X 50 ms pulses (8) or in animals injected with PBS that were electroporated, at 100 V with 5X 50 ms (9). Molecular weight 34 KDa (left) and actin-GFP fusion protein from a Drosophila extract was used as positive control (right). (TIF) [file pone.0263908.s003.tif]

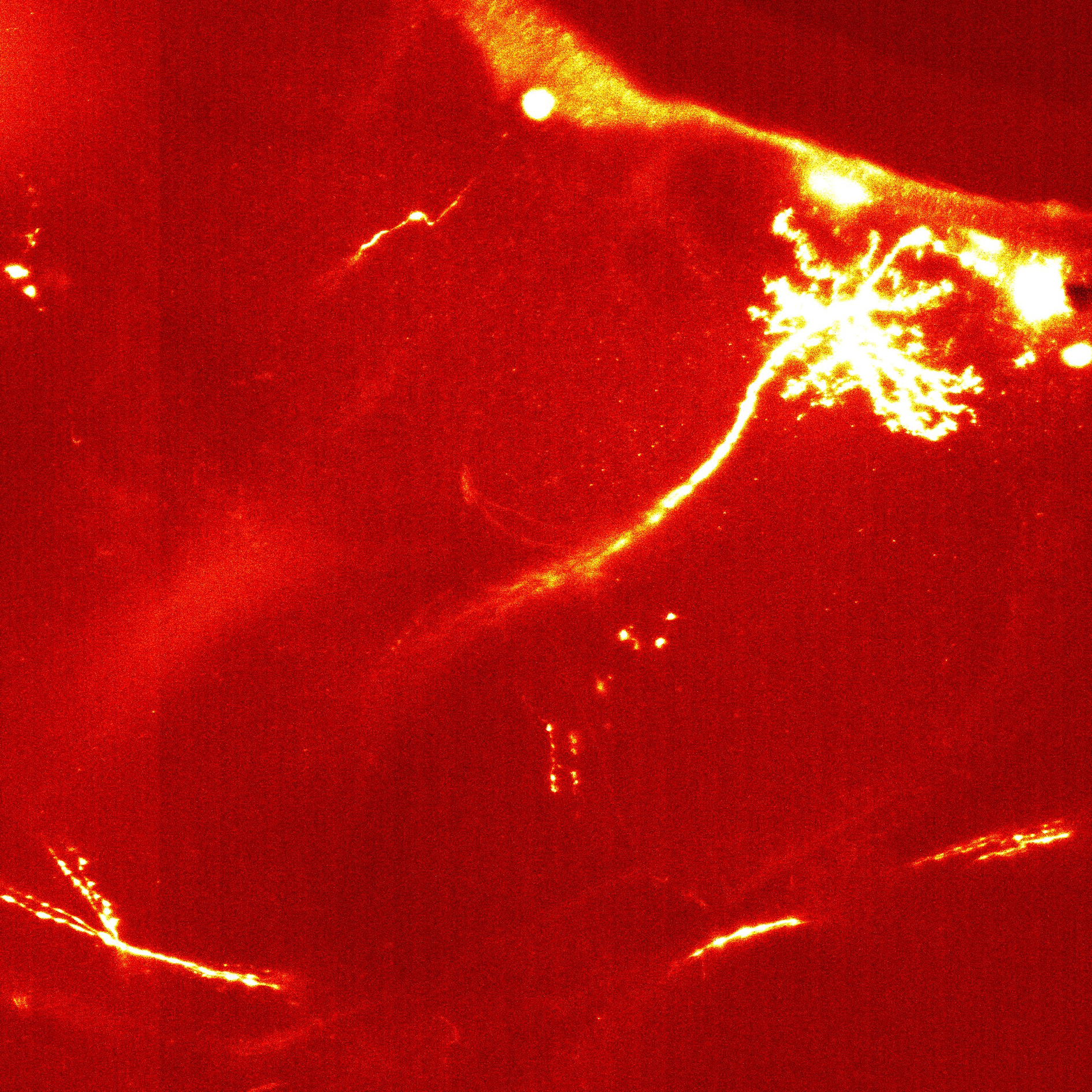

Supplement: S4 Fig — The exposition of the slides of Fig 5 to higher excitation shows the proximal part of the axon connecting the cell body. (TIF) [file pone.0263908.s004.tif]
